# Use of Upadacitinib to Treat a Severe Flare-Up of Rheumatoid Arthritis During Anti-PD-1 Immune Checkpoint Inhibitor Therapy for Stage IV Squamous Cell Carcinoma of the Lung

**DOI:** 10.3390/jcm13206257

**Published:** 2024-10-20

**Authors:** Shunsuke Mori, Kazuyoshi Nakamura, Minori Shimamura, Kouhei Ohe

**Affiliations:** 1 Department of Rheumatology, Clinical Research Center for Rheumatic Diseases, National Hospital Organization (NHO) Kumamoto Saishun Medical Center, Kohshi 861-1196, Kumamoto, Japan; 2 Department of Respiratory Medicine, National Hospital Organization (NHO) Kumamoto Saishun Medical Center, Kohshi 861-1196, Kumamoto, Japan

**Keywords:** immune checkpoint inhibitor, Janus kinase inhibitor, lung cancer, pembrolizumab, rheumatoid arthritis, upadacitinib

## Abstract

**Background:** Immune checkpoint inhibitor (ICI) therapy is becoming the standard of care for the treatment of advanced non-small-cell lung cancer. However, T-cell activation by ICIs frequently induces a flare-up of preexisting autoimmune diseases such as rheumatoid arthritis (RA). Janus kinase (JAK) inhibitors are increasingly used in the treatment of RA, but they could interfere with the efficacy of ICIs by inhibiting interferon signaling. **Case Report:** Here, we describe a case in which upadacitinib, a JAK1-selective inhibitor, was used to manage a severe RA flare-up occurring during ICI therapy with pembrolizumab, an anti-programmed cell death protein-1 antibody. A 54-year-old man with RA was diagnosed with grade IV lung squamous cell carcinoma. The patient had maintained RA remission for 4 years at the time of lung cancer diagnosis. After seven cycles of pembrolizumab therapy, the size of the primary tumor was markedly reduced, but a severe RA flare-up and organizing pneumonia (OP)-like pulmonary lesions occurred. Considering the severity of the flare-up, pembrolizumab was discontinued. Upadacitinib induced swift recovery from the RA flare-up and OP. Eleven months after the last pembrolizumab use, almost all metastatic lesions in the body had disappeared. We did not observe recurrence of lung cancer for more than 1 year during upadacitinib therapy. **Conclusions:** Upadacitinib could be a safe and effective option to treat severe RA flare-ups occurring during anti-PD-1 ICI therapy.

## 1. Introduction

Rheumatoid arthritis (RA) is a chronic autoimmune disease characterized by persistent synovitis and progressive damage to multiple joints [1]. Over the past two decades, significant advances have been made in the management of RA. Immunomodulatory therapies with conventional, biological, and nonbiological targeted disease-modifying antirheumatic drugs (DMARDs) have reduced the disease burden of RA [2]. Given the role of the immune system in tumor surveillance, however, these drugs could increase the risk of developing malignancies. Additionally, RA itself causes disturbances of the immune system, which can affect the risk of certain malignancies [3,4,5].

Lung cancer is one of the most frequently diagnosed cancers worldwide and remains the leading cause of cancer-related death [6]. Recent population-based cohort studies have shown that RA patients are 1.4 to 1.8 times more likely to develop lung cancer compared with the non-RA population [7,8,9,10,11]. In the US Veterans cohort from 2000 to 2017, RA patients had a 23% increased risk of all-cause mortality, and approximately 15% of excess deaths in RA patients were attributed to lung cancer [12]. In our cohort study, the standardized incidence rate compared with the general population was 2.5 for male RA patients, and the risk of lung cancer-related death was 2.5 times higher in RA patients than in non-RA patients between 2006 and 2021 [13,14].

Substantial improvements in our understanding of lung cancer biology at the molecular level have led to the development of new and effective immunotherapies for patients with lung cancer, especially those with non-small-cell lung cancer (NSCLC) [15]. The most progress has been made in immune checkpoint inhibitor (ICI) therapy, which is becoming the standard of care for the treatment of advanced NSCLC. The expression of immune checkpoint proteins, such as programmed cell death protein-1 (PD-1)/programmed cell death ligand-1 (PD-L1) and cytotoxic T-lymphocyte antigen-4 (CTLA-4), downregulates T-cell activation and promotes T-cell apoptosis. This mechanism helps to protect the body from autoimmunity in physiological conditions, but tumor cells utilize it to evade the host immune system [16,17]. Monoclonal antibodies against PD-1, PD-L1, and CTLA-4 are most commonly used as ICIs in the treatment of a wide range of malignancies. In many clinical trials for advanced NSCLC, these ICIs, whether as monotherapy or in combination, have shown a significant survival benefit [18,19].

However, T-cell activation by ICIs frequently induces a broad spectrum of immune-related adverse events (irAEs). In particular, patients with preexisting autoimmune disease are 1.3 to 1.7 times more likely to report irAEs than those without this condition [20,21]. The pooled occurrence rates of irAEs such as flare-ups and new-onset irAEs were 35% to 50% and 23% to 34%, respectively [21,22,23,24]. Most irAEs were manageable with oral glucocorticoid therapy, but some cases required methotrexate (MTX) or biological DMARDs such as tumor necrosis factor (TNF) inhibitors or interleukin (IL)-6 inhibitors [17,25]. Janus kinase (JAK) inhibitors, nonbiological targeted DMARDs, are increasingly used in the treatment of RA [26,27]. However, JAK inhibitors might interfere with the efficacy of ICIs by inhibiting interferon (IFN) signaling [28,29]. The use of JAK inhibitors for the management of flare-ups in RA patients receiving ICI therapy has rarely been reported in the medical literature.

In this study, we report on a case in which upadacitinib, a JAK1-selective inhibitor, was used to manage a severe RA flare-up occurring in a patient receiving ICI therapy with the anti-PD-1 antibody pembrolizumab against stage IV lung squamous cell carcinoma.

## 2. Case Presentation

In August 2011, a 54-year-old Japanese man with a tobacco smoking history of 35 pack-years was diagnosed with seropositive RA. The patient simultaneously developed biopsy-proven organizing pneumonia (OP). The patient exhibited high levels of disease activity for RA, anti-cyclic citrullinated peptide antibodies (anti-CCP Abs), and rheumatoid factor (RF). Pulmonary conditions improved markedly following a 1-month course of high-dose steroid therapy. During the tapering-off of the steroid, MTX monotherapy was introduced as the first-line DMARD for RA. No relapse of OP occurred after the start of steroid tapering. The patient’s RA activity was well controlled thereafter. Seven years later, the patient experienced a flare-up of articular symptoms, but the introduction of peficitinib, a JAK inhibitor, produced favorable outcomes and maintained remission for 4 years. The patient’s health assessment questionnaire (HAQ) score was zero.

**Figure 1:**
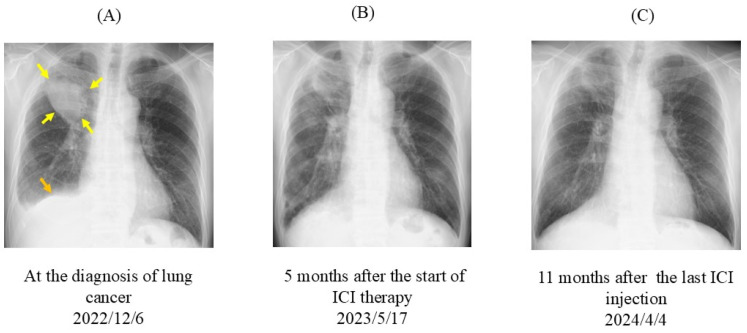
Chest radiographs. (**A**) At lung cancer diagnosis, a large mass (yellow arrows) and pleural effusion (orange arrow) were observed in the right lung. (**B**) Five months after the start of chemotherapy and ICI therapy, the tumor size had reduced. (**C**) Eleven months after the last ICI injection, tumor expansion was not observed. ICI, immune checkpoint inhibitor.

**Figure 2:**
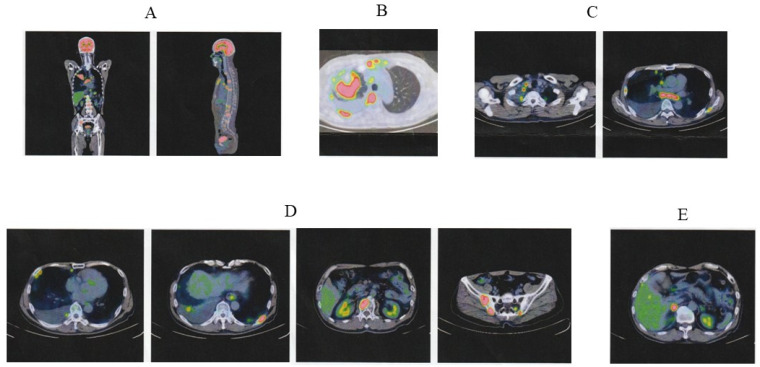
PET-CT images at lung cancer diagnosis. (**A**) Multiple abnormal signals were observed. (**B**) A large mass with strong abnormal uptake was prominent in the right upper lobe of the lung. Abnormal uptakes were also detected in pleural effusion and nodules in the right lung and multiple bones (breastbone and vertebral bodies). (**C**) High metastatic activity was observed in hilar and mediastinal lymph nodes on both sides. (**D**) Multiple bone metastases were detected in vertebral bodies, bilateral ribs, and bilateral iliac bones. (**E**) Abnormal uptake was detected in the right adrenal gland. PET, positron emission tomography; CT, computed tomography; ICI, immune checkpoint inhibitor.

On 6 December 2022, the patient presented with chest pain. Chest radiography revealed a mass (approximately 70 mm × 60 mm in diameter) in the right upper lobe and pleural effusion (Figure 1A). Positron emission tomography-computed tomography (PET-CT) revealed multiple abnormal uptakes (Figure 2). A large solid mass (65 mm in diameter) in the right upper lobe and separate nodules in the right middle and right lower lobes were observed. The maximum standard uptake value (SUVmax) in the pulmonary mass was 11.8. PET-CT also detected metastatic pleural nodules and malignant pleural effusion in the right lung. High metastatic activity was observed in hilar and mediastinal lymph nodes on both sides (SUVmax, 9.8), multiple bones (breastbone, vertebral bodies, bilateral ribs, left scapula, and bilateral iliac bones; SUVmax, 13.7), and the right adrenal gland (SUVmax, 9.3). The clinical tumor-node-metastasis (TNM) stage was IVB (c-T4N3M1ac). Transbronchial biopsy of the pulmonary lesion revealed that the histological type was squamous cell carcinoma. Molecular genetic testing of biopsy samples did not detect epidermal growth factor receptor (*EGFR*) mutations, anaplastic lymphoma kinase (*ALK*) gene rearrangement (fusion), c-ros oncogene 1 (*ROS-1*) gene rearrangement (fusion), or Kirsten rat sarcoma viral oncogene homolog G12C (*KRAS*G12C) mutation. PD-L1 expression according to the tumor proportion score (TPS) was >75%.

**Figure 3:**
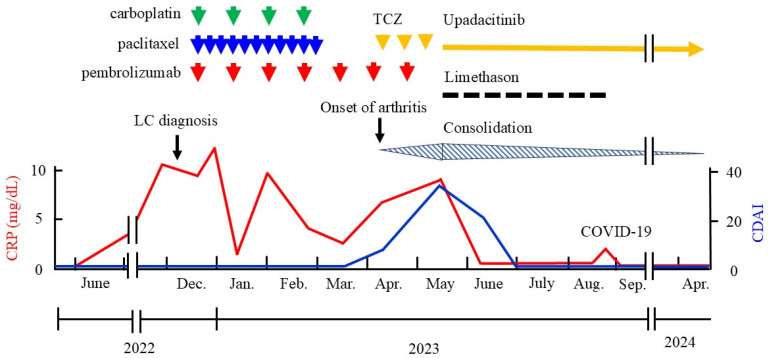
Clinical course with relevant treatment. During upadacitinib therapy, the patient suffered from COVID-19 infection (23 August 2023), but his symptoms were mild. The red line represents serum CRP values and the blue line represents CDAI values. TCZ, tocilizumab; LC, lung cancer; CRP, C-reactive protein; CDAI, clinical disease activity index; COVID-19, coronavirus infectious disease-19.

A clinical course with relevant care is presented in Figure 3. On 21 December 2022, the patient started carboplatin (area under the curve, 5 mg/mL/min on day 1) and nanoparticle albumin-bound paclitaxel (nab-paclitaxel, 100 mg/m^2^ on days 1, 8, and 15) every 3 weeks for four cycles as the first-line chemotherapy, and concomitantly commenced ICI therapy with pembrolizumab, an anti-PD-1 antibody, at 200 mg intravenously every 3 weeks. When ICI therapy was started, the patient’s RA had been in remission for 4 years under peficitinib therapy. Peficitinib was discontinued because of concern that a JAK inhibitor could reduce the efficacy of ICI therapy.

**Figure 4:**
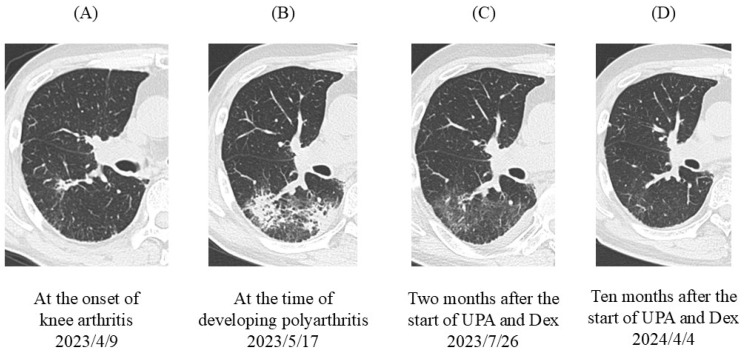
Chest HRCT scans. (**A**) At the onset of knee arthritis, a small consolidation appeared in the right lower lobe (S6). (**B**) At the time the patient developed polyarthritis, patchy consolidations and ground-glass opacities spread from S6 to S10. (**C**) Two months after the start of UPA and Dex therapies, consolidation was improved but ground-glass attenuation remained. (**D**) Ten months after the start of UPA and Dex therapies, abnormal shadows completely disappeared. HRCT, high-resolution computed tomography; UPA, upadacitinib; Dex, dexamethasone.

On 9 April 2023, the patient complained of pain and swelling of the right knee; serum C-reactive protein (CRP) level had increased to 6.5 mg/dL. Along with the occurrence of knee arthritis, a small consolidation appeared in the right lower lobe (S6) (Figure 4A). Sputum was negative for bacterial cultures and polymerase chain reaction (PCR) testing for *Pneumocystis jirovecii*. Antibiotics were not effective. The patient had previously suffered from biopsy-proven OP at RA onset, and OP often follows an RA flare-up [30]. Because RA flare-up and RA-associated OP were suspected, tocilizumab, an anti-IL-6 receptor antibody, was introduced as a monotherapy via subcutaneous injection of 162 mg every other week.

On 17 May 2023, despite the use of tocilizumab, the patient developed polyarthritis with a high clinical disease activity index (CDAI score, 35) and severe functional disability (HAQ score 1.75). Serum CRP was highly elevated (9.1 mg/dL). The results of the synovial fluid test were as follows: Rivalta reaction, positive; total cell count, 12,220/mL; segmented leukocytes, 75%; lymphocytes, 20%; protein, 4.3 g/dL; glucose, 77 mg/dL; and lactate dehydrogenase, 723 U/mL. The consolidation in the right lung spread from S6 to S10 (Figure 4B). The severity of the RA flare-up was grade 3 according to the Common Terminology Criteria for Adverse Events (version 5.0). Limethason, a lipid emulsion containing dexamethasone, was started at a dose equivalent to 2.5 mg of dexamethasone intravenously every 2 weeks (a total of eight times). Tocilizumab was switched to the JAK1 inhibitor upadacitinib (15 mg/day orally). Considering the severity of the RA flare-up, ICI therapy was discontinued; a total of seven cycles were completed. Following upadacitinib therapy, serum CRP was markedly decreased. The patient achieved remission within 6 weeks. Two months later, consolidation was improved (Figure 4C). As of 4 April 2024, the patient’s OP lesions were completely resolved (Figure 4D) and his RA remained in remission.

**Figure 5:**
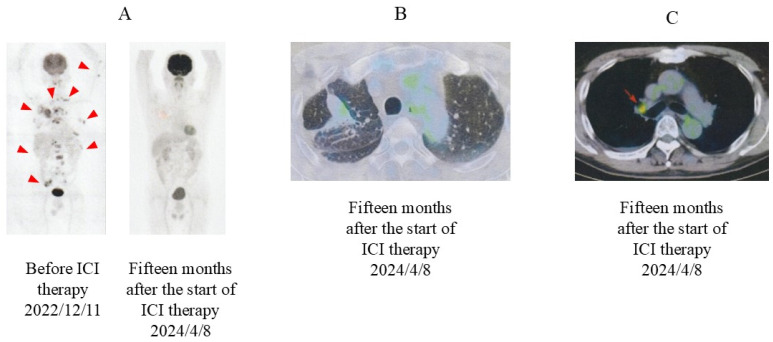
PET and PET-CT images after ICI therapy. (**A**) PET images (not combined with CT), taken before and 15 months after the start of ICI therapy. Before the start of ICI therapy, multiple abnormal signals were observed (red arrowheads). (**B**) PET-CT image taken 15 months after the start of ICI therapy. Abnormal uptake diminished in the primary tumor and disappeared in other multiple lesions. (**C**) PET-CT image taken 15 months after the start of ICI therapy. Residual small uptake remained in the right hilar lymph node (red arrow). PET, positron emission tomography; CT, computed tomography; ICI, immune checkpoint inhibitor.

On 17 May 2023, the size of the primary tumor was markedly reduced on chest radiography (Figure 1B). Eleven months after the last ICI injection, no change in tumor size was observed (Figure 1C). On 8 April 2024, PET-CT revealed that the abnormal signal in the primary tumor had markedly diminished (SUVmax, 2.1) and the separate nodules in the lung had disappeared (Figure 5). Residual small uptake was seen in the right hilar lymph node (SUVmax 3.6), but other abnormal signals were absent.

## 3. Discussion

In the current study, we describe a severe RA flare-up during ICI therapy with the anti-PD-1 antibody pembrolizumab for advanced lung squamous cell carcinoma. The patient had maintained remission for 4 years with peficitinib at lung cancer diagnosis, and this JAK inhibitor was discontinued at the start of ICI therapy. Sixteen weeks later, an RA flare-up and OP-like lesions appeared. Tocilizumab monotherapy failed to control these conditions. The use of upadacitinib together with the intravenous injection of dexamethasone induced the remission of RA within 6 weeks, and OP lesions also improved. Although pembrolizumab was discontinued following the flare-up, the patient had no recurrence of lung cancer for more than 1 year during upadacitinib therapy.

Recent systematic reviews and meta-analyses have shown that RA patients are more susceptible to ICI-related disease flare-ups than patients with other preexisting rheumatic autoimmune diseases: the pooled relative risk is 1.35 [23,24]. In a retrospective cohort study of RA patients with malignancies, McCarter et al. showed that flare-ups occurred in 46% (46 of 100 patients) after starting ICI therapy. In their study, 82% of patients were in remission or had low disease activity at the start of ICI therapy, and 25% were on glucocorticoids and 35% were receiving any DMARDs during ICI therapy. Among these patients, most did not change their anti-RA therapies after starting ICI therapy. Patient characteristics at the start of ICI therapy, including RA disease activity, RA treatment, deformities, and cancer type, were not associated with the risk of flare-up. Most flare-up cases were not severe (grade 1 or 2) and were manageable with oral glucocorticoids. Approximately 20% of patients discontinued ICI therapy following a flare-up [31]. In a single-center retrospective analysis, Efuni et al. showed that RA flare-up occurred in 55% (12 of 20 patients) after the initiation of ICI therapy. When ICI therapy was introduced, 86% of patients had inactive RA, and 73% were receiving glucocorticoids and/or any DMARDs. Patients on glucocorticoid therapies continued their current dose after the start of ICI therapy. Approximately 80% of patients experiencing disease flare-up were successfully treated with oral glucocorticoids, and most patients continued to receive ICI therapy despite flare-up [32]. In our case, the patient had maintained remission for 4 years during peficitinib therapy, and functional disability was not observed at the time of starting ICI therapy. We cannot entirely exclude the possibility that the discontinuation of peficitinib simultaneously with the start of ICI therapy might have resulted in RA flare-up independently of immune activation by ICI therapy. However, the flare-up that occurred after the start of ICI therapy was more severe than a previous flare-up before the development of lung cancer, and it was not resolved by tocilizumab therapy. Although the precise mechanism of irAEs remains unclear, the administration of ICIs leads to aberrant cytotoxic T-cell activation, increased autoantibody production, and production of inflammatory cytokines such as TNF-α, IL-17, and IL-6, which can facilitate the emergence of irAEs [33]. Therefore, targeted inhibition of IL-6 signaling by tocilizumab is considered a rational therapy to treat rheumatic irAEs. In the present case, however, tocilizumab was not effective in the treatment of the RA flare-up following ICI therapy. This can be explained by the idea that disease-associated immune responses involve a complex interaction of multiple cytokines in RA and rheumatic irAEs. Therefore, the blockage of a single cytokine does not necessarily lead to remission in all RA patients [27].

In our case, upadacitinib was effective in managing the RA flare-up occurring after the introduction of ICI therapy. Additionally, the antitumor response continued during upadacitinib therapy, even after discontinuation of ICI therapy. Upadacitinib is a JAK1-selective inhibitor, which interferes with the membrane-to-nucleus signaling of multiple cytokines, such as IL-6, IL-10, IL-2, and type I and type II IFNs, by blocking intracellular JAK-signal transducer and activator of transcription (STAT) pathways [27,34]. Among these cytokines, type I and type II IFNs are considered key mediators in the generation of antitumor action, including direct effects on tumor cells and activation of antitumor immune response [28,35]. In contrast, under conditions of persistent antigen exposure and prolonged IFN signaling, IFNs can have suppressive effects on antitumor immunity. IFNγ, a single type II IFN, has feedback inhibitory effects that avoid the potential toxicity associated with excessive response, which can attenuate antitumor effect. Upon tumor antigen recognition by T cells, prolonged IFNγ production by T cells increases the expression of PD-L1 on tumor cells and tumor-associated macrophages. PD-L1 interacts with its cognate inhibitory receptor PD-1 expressed on tumor-infiltrating T cells, which leads to exhaustion of the T cells and immune evasion of tumor cells. Blocking this negative feedback is a major therapeutic effect of ICI therapy with anti-PD-1 antibodies [29,36,37]. IFNγ upregulates PD-L1 expression on solid tumor cells [38], and the blockade of JAK1 signaling by upadacitinib leads to inhibition of the expression of multiple cytokines (including IFNγ). Thus, it is logical to hypothesize that upadacitinib may play a critical role in the management of ICI-related irAEs. Given the importance of IFNs in the generation of antitumor immune response and the upregulation of PD-L1, however, we were concerned that the use of upadacitinib to treat the RA flare-up might impede the antitumor effect of anti-PD-1 antibody therapy by inhibiting IFN signaling. Nevertheless, recurrence of lung cancer was not observed in our patient during upadacitinib therapy.

Acquired resistance to ICI therapy is common in patients with NSCLC treated with PD-1/PD-L1 blockade. Memon et al. showed an association of chronic and upregulated IFN signaling with acquired resistance in tumors from NSCLC patients who developed resistance to PD-1/PD-L1 blocking therapy [39]. Using in vivo mouse models, Benci et al. showed that prolonged IFNγ signaling in tumor cells can augment the expression of IFN-stimulated genes and ligands for multiple T-cell inhibitory receptors on tumor cells, which increases PD-L1-dependent and PD-L1-independent resistance to immune checkpoint blockage through multiple inhibitory pathways. Both type I and type II IFNs maintain this resistance program. Therefore, blocking IFN signaling via JAK inhibitors can improve immune function of distinct exhausted T-cell populations and restore antitumor responses [40]. In our case, durable antitumor response and RA remission continued for more than 1 year during upadacitinib therapy, which may be explained by this mechanism. In a recent phase 1 clinical trial of the JAK inhibitor ruxolitinib with the anti-PD-1 antibody nivolumab, Zak et al. found that the combination of agents yielded the best overall response rate in patients with Hodgkin lymphoma who were refractory or relapsed after prior ICI therapy. Ruxolitinib was shown to rescue the function of exhausted T cells and enhance the efficacy of ICI blockade in preclinical solid tumor and lymphoma models [41]. Mathew et al. showed that administration of the JAK1 inhibitor itacitinib after ICI therapy with the anti-PD-1 antibody pembrolizumab improved immune function and antitumor response in mice and also resulted in high response rates in a phase 2 clinical trial for metastatic NSCLC. The patients failed to respond to initial ICI therapy with anti-PD-1 antibody but responded well after addition of the JAK1 inhibitor [42]. These studies demonstrated the therapeutic potential of JAK inhibition in combination with anti-PD-1 immunotherapy.

Information about PD-L1 expression levels at the tumor site will contribute to guiding and optimizing PD-1/PD-L1 blocking immunotherapy, especially if this therapy is used in combination with JAK inhibitors. Recently, Mishra et al. reported an accurate and non-invasive PD-L1 quantification tool using PET molecular imaging with two new radiolabeled peptides, which is useful for quantifying total PD-L1 levels at baseline and monitoring accessible PD-L1 levels during therapy. PET monitoring of PD-L1 could provide valuable insights into correlating changes in PD-L1 expression levels with observed responses throughout therapy [43,44].

Murray et al. reported a case in which the use of the pan-JAK inhibitor tofacitinib resulted in rapid and sustained remission from inflammatory arthritis that had occurred after 6 months of ICI therapy with pembrolizumab for lung adenocarcinoma. The patient remained in full remission from arthritis and lung cancer 75 weeks after commencing tofacitinib and discontinuing pembrolizumab [45]. In a recent retrospective observational study, Liu et al. showed that short-term treatment with tofacitinib (for a median of 52.5 days) induced promising clinical efficacy in 47 out of 53 patients (88.7%) experiencing irAEs (including myocarditis, myositis, and hepatitis) during ICI therapies, mainly with anti-PD-1 antibody. ICI therapy was permanently discontinued in 98% of patients, but the antitumor efficacy of ICIs seemed not to be compromised by tofacitinib therapy [46]. Tofacitinib is a first-generation JAK inhibitor, which inhibits JAK1, JAK 3, and partially JAK2. Although peficitinib successfully controlled a previous RA flare-up and maintained remission for 4 years in our case, it is also a non-selective pan-JAK inhibitor. Upadacitinib is a next-generation JAK inhibitor, and thus has higher selectivity than tofacitinib. Considering the risk of unwanted side effects, upadacitinib may be safer for the treatment of RA patients with advanced malignancies [27]. Therefore, in our case, we chose upadacitinib to treat an RA flare-up during ICI therapy for lung cancer. Nevertheless, we should keep in mind that head-to-head direct comparison studies about the safety of different JAK inhibitors are lacking and, therefore, it is not established whether increased selectivity will reduce the risk of malignancies [34].

In the present case, the patient had a tobacco smoking history of 35 pack-years at the time of RA diagnosis. For the treatment of a disease flare-up occurring during MTX monotherapy before the development of lung cancer, we introduced the JAK inhibitor peficitinib. In the oral surveillance study, RA patients aged ≥50 years with one additional cardiovascular risk factor who were receiving tofacitinib 10 mg two times daily had an increased risk for lung cancer compared with those receiving TNF inhibitors. The risk of lung cancer was also increased in current and past smokers who were receiving tofacitinib 10 mg two times a day compared with patients who had never smoked, although these risks were not observed at the currently approved dosage for RA [47]. These data may influence the choice of treatment when the decision is between a JAK inhibitor and a TNF inhibitor, particularly in patients who are at increased risk of lung cancer (i.e., current and past smokers). However, it is not clear whether the increased risk of lung cancer associated with tofacitinib therapy observed in the oral surveillance study may be generalized to other JAK inhibitors [2]. Although it may be hard to justify the use of a JAK inhibitor as a first-line advanced therapy in the majority of RA patients [48], our patient had poor prognostic factors for RA, namely high disease activity and high levels of serum CRP, anti-CCP Abs, and RF, at the first disease flare-up despite long-term MTX therapy. Severe functional disability was also observed. Additionally, the patient desired a DMARD that can be administered orally. In our prospective cohort study, we found that although tofacitinib is an effective treatment option for MTX-refractory RA patients, its effect is significantly lower in patients with previous failure to biological DMARD therapy compared with that in biological DMARD-naive patients [49]. Using our ongoing real-world registries consisting of MTX-refractory RA patients, we also found that tofacitinib can induce greater improvements in biological DMARD-naive patients compared with tocilizumab, but this difference was not observed in previous biological DMARD-failure patients [50]. Considering these points, we chose the JAK inhibitor to treat an RA flare-up that occurred before the lung cancer diagnosis.

## 4. Conclusions

JAK inhibitors are increasingly used in the treatment of RA. Given the role of IFN signaling in upregulating PD-L1 expression, the use of JAK inhibitors might induce tumor resistance to anti-PD-1 antibody by inhibiting IFN signaling. Nevertheless, in the present case, upadacitinib induced the rapid resolution of a severe RA flare-up occurring during anti-PD-1 ICI therapy for advanced lung squamous cell carcinoma. Antitumor response continued during upadacitinib therapy, even after discontinuation of the anti-PD-1 ICI therapy. To date, we have not observed tumor recurrence or RA relapse for more than 1 year in our patient. To our knowledge, this is the first case in which upadacitinib successfully controlled a severe RA flare-up and maintained the antitumor immune response after anti-PD-1 ICI therapy for advanced lung cancer. Upadacitinib can be a safe and effective option to treat severe RA flare-up following anti-PD-1 ICI therapy.

## Data Availability

All data supporting the findings are available from the Human Research Ethics Committee of NHO Kumamoto Saishun Medical Center for interested researchers who meet the criteria for access to confidential data. Because these data include patients’ personal information, the Committee does not recommend that such data be shared publicly. Please contact Mr. Masahiro Hamaguchi, the Control Manager of the Committee, at 616-syol@mail.hosp.go.jp to request data.
